# Are Emotion Regulation Strategies Associated With Visual Attentional Breadth for Emotional Information in Youth?

**DOI:** 10.3389/fpsyg.2021.637436

**Published:** 2021-12-09

**Authors:** Elisa Boelens, Marie-Lotte Van Beveren, Rudi De Raedt, Sandra Verbeken, Caroline Braet

**Affiliations:** ^1^Department of Developmental, Personality, and Social Psychology, Ghent University, Ghent, Belgium; ^2^Department of Experimental Clinical and Health Psychology, Ghent University, Ghent, Belgium

**Keywords:** emotion regulation, attentional breadth, emotion regulation strategies, children, adolescents

## Abstract

Attentional deployment is currently considered as one of the most central mechanisms in emotion regulation (ER) as it is assumed to be a crucial first step in the selection of emotional information. According to the broaden-and-build theory, positive emotions are associated with attentional broadening and negative emotions with attentional narrowing toward emotional information. Given that ER strategies relying on attentional deployment (i.e., rumination, cognitive reappraisal and distraction) have the possibility to influence positive and negative emotions by (re)directing one’s attention, there could be an association with one’s attentional scope. The current study investigated the association between the general (trait) use of three specific ER strategies and visual attentional breadth for positive, negative, and neutral information in a selected sample of 56 adolescents (*M* = 12.54, *SD* = 1.72; 49% girls) at risk for developing psychopathology. First, participants self-reported on their overall use of different ER strategies. Next, the previously validated Attentional Breadth Task (ABT) was used to measure visual attention breadth toward emotional information. No evidence was found for the relationship between 2 specific ER strategies (i.e., cognitive reappraisal and rumination) and visual attentional breadth for neutral, positive and negative emotional information. Surprisingly, “distraction” was associated with visual attentional narrowing, which was unrelated to the valence of the emotion. These unexpected results indicate the multifaceted relationship between trait ER, distraction specifically, and visual attentional breadth for emotional information. Future research, especially in younger age groups, could further elaborate on this research domain.

## Introduction

Since the early 1980s, a growing number of studies have been devoted to the role of emotion regulation (ER) in exploring new research avenues within the field of clinical psychology. ER is often defined as *“the processes by which we influence which emotions we have, when we have them, and how we experience and express those emotions”* (Gross, 1998). Given that the experience of both negative and positive emotions occurs on a daily basis, it is indispensable for an individual to effectively regulate these emotions with the aim of sustaining one’s emotional wellbeing. It is referred to as emotion *dys*regulation when an individual is unable to adequately regulate one’s affect (Izard, 1978; Nezlek and Kuppens, 2008; Grommisch et al., 2019).

The past few decades, emotion *dys*regulation has become a topic of increasing interest as it is linked to 75% of the diagnostic categories of psychopathology described by the Diagnostic and Statistical Manual of Mental Disorders (DSM 5, Kring and Sloan, 2009). Focusing specifically on children and adolescents, emotion *dy*sregulation has repeatedly been associated with symptoms of depression, anxiety, aggressive behavior, and eating- and weight related disorders (e.g., obesity) (Shields and Cicchetti, 1998; Southam-Gerow and Kendall, 2000; Sim and Zeman, 2006; Aldao and Nolen-Hoeksema, 2010; Vandewalle et al., 2016). Because of its strong relationship with a plethora of mental health disorders across different age groups, emotion *dys*regulation has been labeled a transdiagnostic mechanism across psychopathology in both children and adolescents, as well as adults (Kring and Sloan, 2009; Aldao and Nolen-Hoeksema, 2010; Fernandez et al., 2016; Schäfer et al., 2017).

From a developmental perspective, ER is a rapidly and constantly evolving process (Thompson and Goodman, 2010). Early in life, children mostly rely on primary caregivers to externally regulate their emotions (e.g., searching comfort with their parents when being sad or looking at the facial expression of a significant other after falling down) (Morris et al., 2007). Still, studies have shown that infants and pre-schoolers are also able to *autonomously* regulate basic emotions (e.g., anger and fear) through the use of behavioral strategies (e.g., playing with toys to distract themselves or cuddle with their favourite bear to comfort themselves) (Kopp, 1982; Eisenberg and Morris, 2002). As cognitive and emotional functioning matures, children additionally start to use more sophisticated cognitive strategies to internally and independently regulate their emotions (e.g., re-evaluation or acceptance of the situation) by the age of eight to nine (Harris, 1989). Although the development of ER appears linear from this point of view, research shows a maladaptive shift in ER during the adolescent phase due to the heightened emotional reactivity and various stressors characterizing this developmental period. This shift is characterized by a general decrease in the use of adaptive ER strategies such as problem solving and cognitive reappraisal and an overall increase in maladaptive strategies such as rumination and aggression (Cracco et al., 2017). Given this remarkable shift in ER, studying ER in adolescence is of key importance. Moreover, such research should pay attention to gender differences in ER as remarkable differences occur from childhood to adolescence under the influence of biological and contextual factors, i.e., girls are generally better at regulating their emotions than boys in childhood, whereas boys appear to do better in adolescence (Morris et al., 2002; Cracco et al., 2017).

Differences can be observed in the specific ways individuals regulate both positive and negative emotions using various ER *strategies* (Weiss et al., 2019). Besides ER strategies for regulating positive emotions (for review see Carl et al., 2013), this study particularly focuses on ER strategies for regulating negative emotions. Next, a distinction is often made between adaptive and maladaptive ER strategies – based on their general (trait) and long-term associations with psychopathology (Aldao and Nolen-Hoeksema, 2012). Adaptive ER strategies lead to a decrease in negative affect (and conversely a restoration or an increase in positive affect), as well as greater levels of psychological well-being in the long-term (Aldao and Nolen-Hoeksema, 2012). Some renowned adaptive ER strategies for regulating negative emotions in children and adolescents are distraction, cognitive reappraisal, problem solving, and acceptance (Cicchetti, 2010; Werner and Gross, 2010; Aldao et al., 2014). On the flipside, maladaptive ER strategies are associated with an increase in negative and a decrease in positive affect and is associated with more overall psychopathology in the long-term (Gross, 2002; Aldao et al., 2010; Werner and Gross, 2010). Examples of maladaptive ER strategies for regulating negative emotions used by both adults, children and adolescents are avoidance, rumination and suppression (Aldao et al., 2010; Aldao and Nolen-Hoeksema, 2010, 2012). Although this categorization has proven its validity in previous research (e.g., Aldao et al., 2014; Schäfer et al., 2017), recent studies point out that when it comes to the use of these strategies in daily life, a more nuanced approach should be considered for the evaluation of the adaptiveness of an ER strategy, taken into account numerous other co-determining factors (e.g., the context in which these strategies are used, the flexibility with which they are employed) (Aldao et al., 2015).

Initially, ER was conceptualized as a one-dimensional process that solely involved the control or elimination of negative emotions. Throughout time, it has evolved toward a multidimensional construct in which different cognitive, attentional and behavioral processes are considered (Cole et al., 2004; Gratz and Roemer, 2004; Rajappa et al., 2012). One of the leading frameworks describing the emotion regulatory processes is the ‘process model’ of Gross (1998). The model largely distinguishes between five groups of regulation processes that are placed on a temporal dimension being (1) *situation selection* or the ability to influence emotions by selecting or avoiding specific situations in which emotions can occur, (2) *situation modification* or the ability to influence emotions by changing external, physical characteristics of the situation, (3) *attentional deployment* or the process of directing the attention in a particular way *within* a situation in order to influence the situation-specific evoked emotions, which can take many forms including *physical withdrawal of attention* (e.g., covering the eyes), *internal redirection of attention* (e.g., redirecting your thoughts) and *external redirection of attention* (e.g., someone pointing something out), (4) *cognitive change* or the ability to influence emotions by reappraising the emotion-eliciting situation, and lastly (5) *response modulation* or the ability to influence physiological and/or behavioral responses associated with the emotion. It is generally assumed that problems in ER can occur in each of the phases in the ER process (Gross and Thompson, 2007; Koole, 2009).

In contrast to situation selection and/or situation modification, attentional deployment is a regulatory process through which emotions can be controlled without modifying or changing the situation itself. Gross and Thompson (2007) state that attentional processes may be a fundamental mechanism of (a) the emotion generative and (b) the emotion regulation process. More specifically, attentional deployment can be considered a crucial first step in the selection of emotional information and influence the ability to engage in specific ER strategies, but also serves as a central mechanism within certain ER strategies (i.e., cognitive reappraisal) (Gross and Thompson, 2007; Koole, 2009; Wadlinger and Isaacowitz, 2011; Todd et al., 2012).

Competencies and deficits in attentional deployment show many individual differences. Competencies continue to develop throughout childhood and become more prominent with age (Fox and Calkins, 2003; Ochsner and Gross, 2005; Gross and Thompson, 2007; Godara et al., 2020). As early as the age of five, attentional deployment is considered an important regulatory skill (e.g., children distract their selves when being separated from their mother by deploying their attention away trough play) (Sethi et al., 2000; Mischel and Ayduk, 2004). As individuals grow older and reach early adolescence, they become more aware of how they can autonomously manage their emotions by deploying their attention away and will eventually be using even more (complex) strategies that rely on this process (e.g., coping with a fearful situation by taking in the information and consequently deploying attention toward alternative interpretations of the event) (Gross, 2013; Ahmed et al., 2015).

Deficits in attentional deployment can occur at an early age. In one study conducted in 7-year-old clinically anxious children, results showed that there was a significantly larger attentional focus on emotional information (i.e., angry faces) in clinically anxious children compared to children with no or lower levels of anxiety symptoms (Taghavi et al., 1999; Waters et al., 2008). However, evidence on deficits in attentional deployment is largely limited to adults (e.g., Power and Dalgleish, 2015). In general, these studies show that the onset and maintenance of different forms of psychopathology (e.g., depression and anxiety) is associated with the navigation of attention toward negative emotional information, resulting in the maintenance or intensification of concurrent negative emotional states.

Given that both deficits in attentional deployment and ER more broadly are associated with psychopathology, studies unraveling the association between attentional in an emotional context and ER strategy use can provide crucial information for both research and clinical practice (Fox and Calkins, 2003; Ochsner and Gross, 2005; Gross and Thompson, 2007; Godara et al., 2020). Nevertheless, only few studies regarding these associations have been conducted, especially in younger age groups. One study using a visual attentional probe task tentatively suggests that children who are able to control their attention and consequently prevent themselves from focusing on negative information, generally use more adaptive ER strategies and report low levels of psychopathology (Waters et al., 2008). Furthermore, a recent experimental study using a visual attentional breadth task in a selected sample of adolescents (Van Beveren et al., 2020), investigated the link between visual attention across neutral and emotional information contexts and ER in (early) adolescents. Participants were presented various Emoji (i.e., positive-, negative-, and neutrally valenced) and were afterward instructed to correctly identify peripheral information (= dependent variable) on a computer screen (i.e., detect a black circle that was simultaneously presented close or far from the Emoji). Results showed that the general (trait) use of adaptive ER strategies was associated with a broadened attentional scope for neutral (but not for positive or negative) information. This in turn was related to more positive affect and overall emotional well-being (Fredrickson and Branigan, 2005; Van Beveren et al., 2020). However, additional research is pivotal to further invigorate these findings, especially in at risk youth.

## Emotion Regulation Strategies and Attentional Scope

Common trait ER strategies that predominantly rely on attentional deployment, and may therefore be associated with one’s attentional scope in an emotional context, are cognitive reappraisal, rumination and distraction (Gross, 2013).

### Cognitive Reappraisal

Cognitive Reappraisal is generally categorized as an adaptive ER strategy and refers to the way in which a positive perspective or reinterpretation of the situation can decrease negative affect and/or increase positive affect (McRae et al., 2012; Van Cauwenberge et al., 2017). In general, cognitive reappraisal has been linked to the fourth phase of the ER process (i.e., cognitive change). However, more recent studies (e.g., Strauss et al., 2016; Sanchez-Lopez et al., 2019) show that the quality of reappraisal depends on the earlier process of attentional deployment. More specifically, the ability to focus on negative emotional information, followed by disengaging the attention away from this information and shifting the attention toward other, more positive or neutral, emotional information, is crucial for the successful use of this ER strategy. In both youth and adults, an underutilization or ineffective use of cognitive reappraisal has been associated with higher depressive and anxiety symptoms (Aldao et al., 2010; Dryman and Heimberg, 2018).

### Rumination

Rumination is considered a maladaptive ER strategy in which there is a repetitive focus on (negative) emotions, its causes, as well as its consequences (Nolen-Hoeksema et al., 2008; Gross, 2013). Rumination involves directing the attention toward the negative emotion(s) but is, in contrast to cognitive reappraisal, accompanied with negative beliefs (e.g., negative emotions are unacceptable) (Gross, 2013). Rumination has mainly been associated with internalizing problems such as depression and anxiety in both youth and adult populations (Aldao et al., 2010).

### Distraction

Distraction represents the intentional shift in attention from one’s negative emotions toward an external situation or stimulus (Gross, 1999; Scheibe et al., 2015) and has been found to be an adaptive ER strategy across various studies (Sheppes et al., 2011). However, research shows that distraction can have a rather ambiguous effect on emotions and emotional well-being (Gratz and Tull, 2011; Craske and Barlow, 2014). When distraction is used to completely avoid negative emotions, instead of being used in a flexible and adaptive way to redirect the attention away, it may have limited short-term advantages and even adverse long-term consequences (Gross, 1998; Van Dillen and Papies, 2015; Wolgast and Lundh, 2017).

According to the broaden-and-build theory of positive emotions (Fredrickson, 1998, 2001), attention can be (re)directed. This means that the attentional scope, which refers to a range of thoughts, perceptions, and actions that occur in a certain situation, can be narrowed or broadened (i.e., *attentional breadth*) (Whitmer and Gotlib, 2013). A broadened attentional scope is associated with the intake of peripheral stimuli and global information (e.g., seeing a barking dog but also noticing it is on a leash and the owner is with him) whereas a narrowed attentional scope is associated with processing only central stimuli and local information (e.g., focusing solely on the sharp teeth of the dog) (Sung and Yih, 2016; Wronska et al., 2018).

In this theory it is stated that specifically the attentional scope related to negative and positive emotions has different effects on both cognition and psychophysiology. Studies have shown that positive emotions (e.g., optimism and happiness) are associated with a more broadened attentional scope, which is considered a protective factor against stressful events (Fredrickson and Joiner, 2002; Rowe et al., 2007). Negative emotions (e.g., anger and anxiety) however, are associated with a more narrowed attentional scope which is related to emotional disorders (e.g., anxiety) (Fredrickson and Joiner, 2002). Although the mechanism behind this process is still under study, attentional narrowing is often seen as an evolutionary threat-driven response that is beneficial when it leads to quick and decisive action. Nowadays, feelings of stress-related anger, and anxiety are often not related to real life-threatening stressors. Yet, the response remains the same. As a result, the disproportional and continuous reactivity in response to stress that is characterized by narrowed attention often leads to maladaptive and rigid action (e.g., aggressive actions) and thinking patterns (e.g., ruminating about the stressful event), which eventually can cause emotional problems (Gasper and Clore, 2002; Gasper, 2004; Yoon et al., 2015; Gu et al., 2017).

Based on the broaden-and-built theory of positive emotions (Fredrickson, 2001), it can be assumed that particularly ER strategies that rely on the process of attentional deployment (i.e., cognitive reappraisal, rumination, distraction) will be associated with the breadth of one’s attentional scope. As the goal of adaptive ER strategies such as cognitive reappraisal is to restore/increase positive and decrease negative affect, it is plausible to assume an association with visual attentional *broadening* (Aspinwall, 1998; Fredrickson and Joiner, 2002), whereas for maladaptive ER strategies such as rumination an association with visual attentional *narrowing* is theoretically presumed (Nolen-Hoeksema et al., 2008).

To our knowledge, no existing study provides direct evidence for the relationship between the attentional scope and two specific ER strategies (i.e., cognitive reappraisal, and distraction). Based on the aforementioned line of thought and existing literature (see e.g., Sanchez-Lopez et al., 2019), it is to be expected that cognitive reappraisal is associated with a broadened attentional scope as this strategy involves the ability to take in negative emotional information and subsequently shift attention toward more positive or neutral information (Strauss et al., 2016). Given that distraction involves actively shifting the attention away from negative emotional information (Scheibe et al., 2015), one could expect an association with a broadened attentional scope toward emotional information. However, as distraction is considered a rather *ambiguous* ER strategy that becomes *un*helpful when used in the long run to avoid negative emotions, an association with a narrowed attentional scope toward emotional information is also plausible (Förster et al., 2006). Lastly, based on the attentional scope model of rumination (Whitmer and Gotlib, 2013) which states that rumination involves a perseverative focus on negative emotional information, an association between rumination and attentional narrowing is to be expected (Grol et al., 2015; Fang et al., 2017). Unfortunately, evidence for these theoretical propositions is scarce and limited to adults.

## The Current Study

The purpose of the current study is to examine the relationship of the general (trait) use of specific ER strategies and the attentional scope in youth (9–15 years) in an experimental study. The broaden-and-build theory of Fredrickson (1998, 2001) states that positive emotions are associated with attentional broadening and negative emotions with attentional narrowing (Aspinwall, 1998; Fredrickson and Joiner, 2002; Rowe et al., 2007). Given that ER has the possibility to influence both positive and negative emotions which could be related to a broadened or narrowed attention scope (Aspinwall, 1998; Fredrickson and Joiner, 2002), and the fact that certain specific ER strategies (i.e., cognitive reappraisal, rumination, and distraction) greatly rely on attentional deployment, it is hypothesized that (1) the use of the adaptive strategy “cognitive reappraisal,” in children and adolescents will be associated with a broadened attentional scope, (2) the use of the maladaptive strategy “rumination” will be associated with a more narrowed attentional scope (Grol et al., 2015; Fang et al., 2017), and (3) that the use of the ambiguous ER strategy “distraction,” in children and adolescents, will either be associated with narrowed attention or with attentional broadening. Because of age and gender differences in ER, these differences will be considered in all analyses (Hyde et al., 2008).

## Materials and Methods

### Participants

Fifty-six youth between 9 and 15 years (*M* = 12.54, *SD* = 1.72; 49% girls) were recruited (see Table 1). All youth were admitted to a residential treatment centre for a multidisciplinary obesity treatment [MOT; (Braet and Van Winckel, 2001)]. Although obesity is considered a pathology rather than a type of psychopathology, previous research uncovered large co-morbidities with psychological problems such as depressive symptoms, low self-esteem, and behavioral problems (Braet et al., 1997; Strauss, 2000). Recent studies in youth highlight the association between the transdiagnostic mechanism emotion *dys*regulation and emotional and/or external eating (Harrist et al., 2013). Especially an underutilization of adaptive (i.e., reappraisal) as well as a frequent use of maladaptive ER strategies (e.g., rumination) is linked to emotional eating and seems to play a crucial role in eating- and weight-related pathology and related psychological problems (Kubiak et al., 2008; Evers et al., 2010; Vandewalle et al., 2016). Given the association between these strategies and attentional deployment, a more detailed study can give new insight in the development and occurrence of emotional problems within this sample (Gross, 2013). In the current study the Child Depression Inventory (CDI; Kovacs, 1992; Timbremont and Braet, 2002) was used to screen for cognitive, affective, and behavioral symptoms of depression. To interpret the total score of the questionnaire, a cut-off score of 12 was used to indicate the presence of mild to moderate symptoms of depression, a score above the cut-off of 16 indicates severe symptoms of depression. In current sample a mean score of 13.70 (*SD* = 6.54) ranging from 1 to 29 was found. Since the current study is part of a larger project (Debeuf et al., 2020) on studying mechanisms of ER and the effects of training ER in children and adolescents with obesity, this sample can be referred to as a convenient sample of youth at risk for developing psychopathology.

**TABLE 1:** Frequency and percentage of age and gender.

Age	N *_Total_*	%	N*_boys_*	%_boys_
9	1	2.4	1	100
10	6	14.6	3	50
11	5	12.2	1	20
12	7	17.1	5	71.4
13	7	17.1	6	85.7
14	10	24.4	4	40
15	5	12.	1	20
**Total**	**41**	**100**	**21**	**51.2**

In the current study, inclusion criteria were used. First, youth could not be enrolled in the inpatient treatment for more than two months. Second, youth should master Dutch and/or French. All youth and their parents were informed about the procedure and their right regarding GDPR^1^ prior to the study and assented on taking part. Participation was not remunerated.

### Procedure

The research protocol was approved by the Committee of Medical Ethics (EC UZG 2018/0101). After obtaining informed consent, participants were asked to fill out a self-reported trait emotion regulation (i.e., FEEL-KJ) and a depression (i.e., CDI) questionnaire using an online platform hosted by the Research Unit on a computer of the treatment center (see measures below) prior to a lab-study that took place on the same day. On the day of testing, adolescents were instructed to first explore the lab with the aim of familiarizing themselves with the lab-setting. As previously mentioned, the current study was part of a larger experimental study, took approximately 45 min per participant and consisted of several phases. Adolescents completed an attentional breadth task (see measures below) after watching a white screen for 3 min, which served as a neutral baseline to prevent experiencing negative affect at the start of the experimental task (Patuzzo et al., 2003). After completing the experimental task for ± 40 min, children and adolescents were verbally instructed to complete an additional 5 min abdominal breathing exercise in order to ensure participants left the lab in a good state of mind (Verbeken et al., 2019). Youth self-reported on fluctuations in affect at 4 different time points during the experiment (see measures below).

### Measures

#### Self-Report Questionnaires

##### Emotion Regulation Strategies

To assess the general use of ER the *Fragebogen zur Erhebung der Emotionsregulation bei Kindern und Jugendlichen* (FEEL-KJ); (Grob and Smolenski, 2005; Braet et al., 2013) was used. This questionnaire is used in children and adolescents between 8 and 18 years old and measures the way children and adolescents regulate feelings of anger, sadness, and anxiety. In total 90 items measure 15 specific ER strategies that can be categorized as adaptive (e.g., cognitive reappraisal), maladaptive (i.e., rumination), or external ER strategies (i.e., expression). These strategies are measured as trait ER strategies (i.e., the general use of these strategies when experiencing negative affect). For each of the emotions (i.e., anger, sadness, anxiety), the same 30 items are presented on a 5-point Likert scale (1 = almost never, 2 = rarely, 3 = occasionally, 4 = often, 5 = almost always). An example item is: “*When I am sad/angry/anxious, I accept what makes me angry”* A total score can be calculated for each of the strategies (measured with 2 items for each of the 3 emotions; scores ranging between min 0.6 and max 0.30), in which a higher score means the ER strategy is more often used. In addition, a total score can also be calculated for the total use of adaptive, maladaptive, and external ER. The FEEL-KJ is considered reliable and valid with an acceptable to good internal consistency over all subscales (Cronbach’s alpha between 0.64 and 0.94). Furthermore, an acceptable to good test-retest reliability was reported with correlation coefficients between 0.76 and 0.90 (Cracco et al., 2015). In the current study, we focused on three strategies relying on attentional deployment: (1) cognitive reappraisal (FEEL-KJ-CR), (2) rumination (FEEL-KJ-RUM) and (3) distraction (FEEL-KJ-DIS). Each of the strategies was assessed with six items (two items per emotion), e.g., “*When I’m sad/angry/anxious, I tell myself that it is not that bad”* to measure cognitive reappraisal; “*When I’m sad/angry/anxious, I can’t get this out my mind”* to measure rumination*;* “*When I’m sad/angry/anxious, I do something I like”* to measure distraction. Cronbach’s alphas reveal acceptable to good internal consistency of 0.74, 0.72, and 0.81 for the three specific strategies.

##### Affective States

To measure fluctuations in affect during the lab study, the intensity of positive and negative affect was obtained through Visual Analog Scales (VAS). Youth scored their feelings of happiness, sadness, anxiety, frustration, boredom, and anger on a scale from zero (not present) to a hundred (very present) (Aitken, 1969; Bond and Lader, 1974). This was measured on four different time points, i.e., before (1) and after (2) the neutral baseline, after (3) the Attentional Breadth Task and after (4) the breathing exercise.

#### Experimental Task

##### Attentional Breadth Task

**FIGURE 1:**
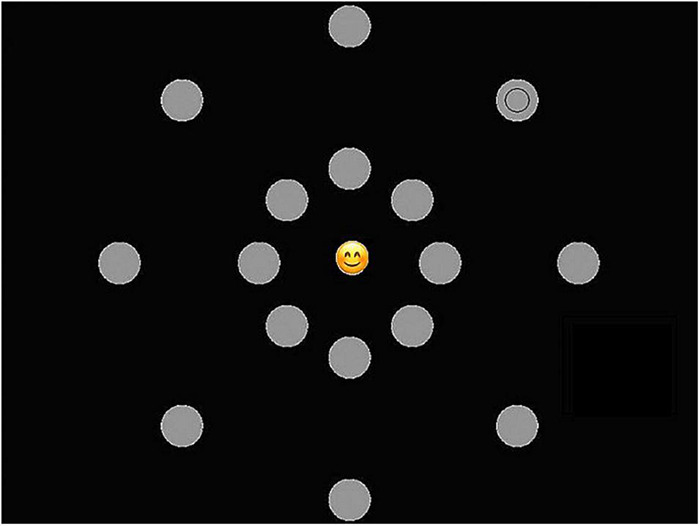
Central Emoji and target stimulus of the attentional breadth task. Note: based on Van Beveren et al. (2020).

Visual attentional breadth in relation to centrally presented emotional stimuli was measured using an experimental task (Bosmans et al., 2009). The task has successfully been used in different studies to measure fluctuations in attentional broadening/narrowing related to centrally presented, relevant information (Bosmans et al., 2009; Grol and Raedt, 2014; Grol et al., 2015). Recently Van Beveren et al. (2020) adapted and evaluated this task using child friendly emotional stimuli (i.e., Emoji) to measure fluctuations in visual attentional breadth for emotional information in (early) adolescents. Participants were seated at a distance of 10.63 inches from a 19″ CRT computer screen, using a chin rest to guarantee correct positioning and distance to the screen. In each trial a picture of an Emoji (82 × 82 pixels) appeared in the centre of the screen (central Emoji; see Figure 1). The Emoji could be categorized as negative, positive, or neutral and eight validated Emoji were selected for each category. The selection of Emoji was based on both valence (i.e., negative and positive) and arousal (i.e., low arousal and high arousal) and was evaluated in previous research within a comparable age and gender sample of (early) adolescents (i.e., only Emoji that were clearly identified as either positive, negative or neutral are included) (Vanden Berghe et al., 2020).

**FIGURE 2:**
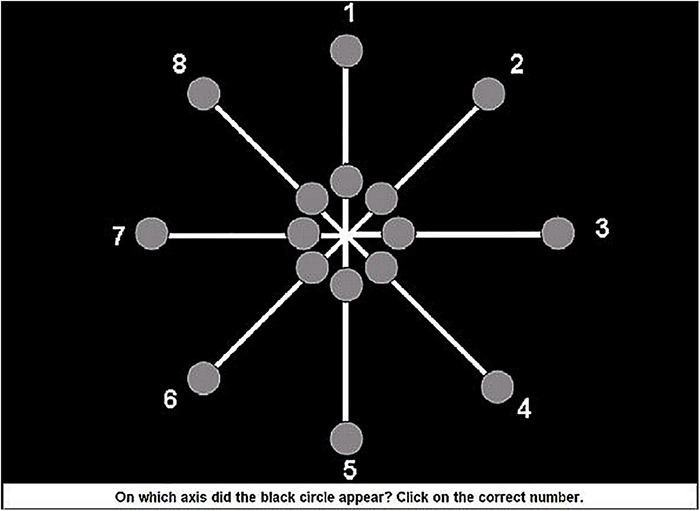
Response screen axe of the target stimuli. Note: based on Van Beveren et al. (2020).

**FIGURE 3:**
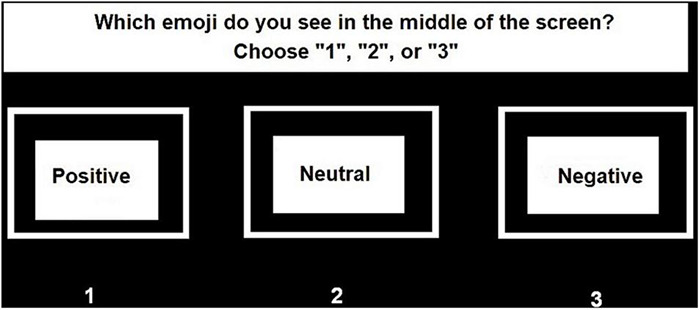
Response screen valence of the central Emoji. Note: based on Van Beveren et al. (2020).

When the central Emoji appeared, 16 gray dots with a diameter of 2 cm simultaneously surfaced in two concentric circles around the Emoji (see Figure 1). In total 8 imaginary axes appeared around the Emoji with two grays dots on each axe (compare Figures 1–3). One of the two gray dots appeared at 4.41 inches (i.e., far) from the central Emoji at 25° of the visual angle, the other gray dot appeared at 1.77 inches (i.e., close) from the central Emoji at 10° of the visual angle. At the same time of the presentation of both the emoji and gray dots, a smaller black circle with a diameter of 0.51 inches appeared in one of the 16 gray dots (target stimuli; see Figure 1). The black circle could thus be close or far from the Emoji. In order to prevent confounds of saccadic eye movements in search of the peripheral target (Ball et al., 1988), all stimuli were presented for 68 ms.

Participants were instructed to (1) correctly identify the valence of the presented central Emoji (i.e., negative, positive, and neutral), and (2) localize the black circle that appeared in one of the 16 gray dots. For both responses there was no time limit. After the presentation of the central Emoji and target stimuli, a first screen appeared and participants were asked to identify the valence of the central Emoji (see Figure 2). After giving this response, a second screen appeared after which participants immediately were asked to identify on which of the eight axes the target stimuli had appeared (see Figure 3). In the current study the main dependent variable was the accuracy on localizing the peripheral target stimuli. This was calculated based on trials in which the participants also correctly identified the valence of the central Emoji to make sure the participants maintained attention to the center of the screen during the task.

The task consisted of eight practice trials with a 250 ms presentation time, followed by eight practice trials with a shorter 68 ms presentation time. The test phase itself consisted of 144 trials with six types of trials randomly presented in three blocks of 48 trials each. The type of trials were based on the distance of the target stimuli to the central Emoji, as well as their valence (i.e., positive close, positive far, neutral close, neutral far, negative close, and negative far).

#### Data-Analytic Plan

For the analyses of the attentional breadth task, all trials were deleted in which the central Emoji was incorrectly identified, to make sure participants also maintained attention to the centre of the screen during the task. The dependent variable was the number of correctly identified target stimuli (black circle). Only trials with correctly identified central Emoji were included. Performance on the attentional breadth task was examined by performing a 3 Valence (Positive vs. Neutral vs. Negative) × 2 Distance (far vs. close) mixed ANOVA with the accuracy rates, i.e., number of correctly localized peripheral targets as the dependent variable.

Thereafter we calculated an Attentional Narrowing Index (ANI) by subtracting the proportion of correctly identified targets in the far trials from the proportion of correctly identified targets in the close trails for positive trials (posANI), neutral trials (neuANI), and negative trials (negANI) separately. Although our primary interest lays in attentional broadening, we calculated ANI scores to keep the outcome variables consistent and to enable comparison with the original task used in previous research (Bosmans et al., 2009; Grol and Raedt, 2014; Grol et al., 2015; Van Beveren et al., 2020). Higher ANI scores imply that more distance from the central picture leads to less correct answers and therefore can considered to be a measure of attentional narrowing/breadth. Next, gender and age effects were added as covariates into the model. Finally, we ran three additional 3 Valence (Positive vs. Neutral vs. Negative) × 2 Distance (Far vs. Close) mixed ANOVAs with FEEL-KJ-DIS, FEEL-KJ-RUM, and FEEL-KJ-CR as continuous predictors to test our research questions.

## Results

### Preliminary Analyses and Group Characteristics

In total, on average 14.58% of the trials was deleted due to incorrect identification of the central Emoji. Participants (*n* = 15) were excluded from further analysis if the number of deleted trials for any of the different trial types was more than 50% (Grol and Raedt, 2014). Descriptive statistics for all study variables and bivariate correlations amongst these variables are presented in Table 2.

**TABLE 2:** Descriptive statistics and bivariate correlations in the final sample.

	M (*SD*)	min – max	FEEL-KJ-CR	FEEL-KJ-RUM	FEEL-KJ-AS	FEEL-KJ-MS
Age	12.54 (1.72)	9 – 15				
FEEL-KJ-DIS	43.70 (10.01)	22 – 63	0.391*	0.019	0.786**	–0.230
FEEL-KJ-CR	47.39 (10.45)	28 – 71		0.336*	0.680**	0.369*
FEEL-KJ-RUM	49.98 (12.42)	27 – 80			0.230	0.758**
P-POS-close	0.48 (0.30)	0.00 – 0.96				
P-POS-far	0.22 (0.17)	0.00 – 0.70				
P-NEU-close	0.47 (0.26)	0.04 – 0.96				
P-NEU-far	0.23 (0.14)	0.04 – 0.50				
P-NEG-close	0.46 (0.27)	0.00 – 0.96				
P-NEG-far	0.21 (0.14)	0.00 – 0.71				

FEEL-KJ-RUM = self-reported emotion regulation strategy “rumination,” FEEL-KJ-CR = self-reported emotion regulation strategy “cognitive reappraisal,” FEEL-KJ-DIS = self-reported emotion regulation strategy “distraction.” P = proportion of correctly localized peripheral targets presented far or close when the central stimulus was positive (POS), neutral (NEU), or negative (NEG). *p < 0.05; **p < 0.01.

A non-parametric Friedman’s ANOVA was performed as the data on the percentage of deleted trials was non-normally distributed. The ANOVA test revealed that if the target stimulus was presented close to the central stimulus, there were significant differences in the accuracy for identifying the central stimulus depending on the *emotional valence*, χ^2^ (2, *N* = 41) = 33.90, *p* < 0.001. Follow-up Wilcoxon’s signed-rank tests showed that participants made less errors when identifying neutral compared to positive stimuli (*Z* = –4.31, *p* < 0.001), as well as neutral versus negative stimuli (*Z* = –4.39, *p* < 0.001) when the target stimulus was presented close to the central stimulus. No difference between negative and positive stimuli was detected (*Z* = –0.29, *p* = 0.769).

A second ANOVA test revealed that, if the target stimulus was presented far from the central stimulus, significant differences occurred in accuracy for identifying the central stimulus depending on *the emotional valence*, χ^2^ (2, *N* = 41) = 34.85, *p* < 0.001. Again, follow-up Wilcoxon’s signed-rank tests showed that participants made less errors when identifying neutral compared to positive stimuli (*Z* = –4.29, *p* < 0.001), as well as neutral versus negative stimuli (*Z* = –4.50, *p* < 0.001) when the target stimulus was presented far from the central stimulus. No difference between negative and positive stimuli was detected (*Z* = –0.96, *p* = 0.338).

### Task Performance

**FIGURE 4:**
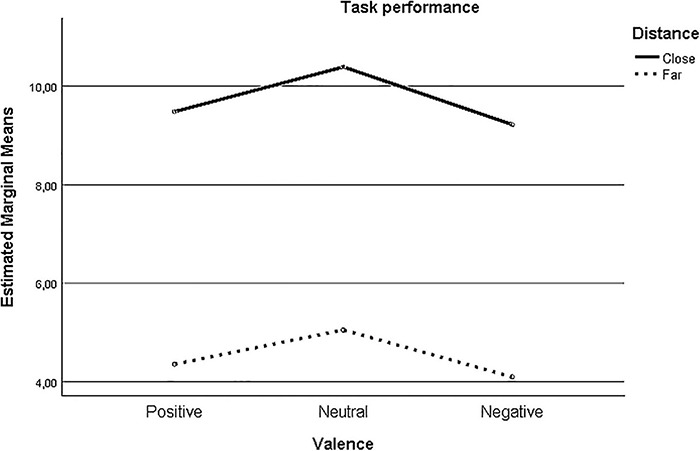
Task performance. Note: estimated marginal means for the 3 Valence (Positive vs Neutral vs Negative) × 2 Distance (Far vs Close) interaction on the number of correctly localized target stimuli are displayed on the *y*-axis.

The 3 Valence (Positive vs. Neutral vs. Negative) × 2 Distance (Far vs. Close) mixed ANOVA on the accuracy rates of target detection (see Figure 4) yielded a main effect of Distance, *F* (1, 40) = 67.80, *p* < 0.001, $\eta_{p}^{2}$ 0.63, indicating that the number of correct identifications of the target stimulus was significantly higher when the target stimulus appeared close compared to far from the central stimulus (*M*diff = 5.20, *SE* = 0.63, *p* = 0.001), and a main effect of Valence, *F* (2, 80) = 6.19, *p* = 0.003, $\eta_{p}^{2}$ 0.13, indicating that the number of correct identifications of the target stimulus was higher when the target stimulus was neutral compared to positive (*M*diff = 0.78, *SE* = 0.335, *p* = 0.025) or negative (*M*diff = 1.06, *SE* = 0.25, *p* < 0.001), but not when the target stimulus was positive compared to negative (*M*diff = 0.280; *p* = 0.417). Finally, no significant 3 Valence (Positive vs. Neutral vs. Negative) × 2 Distance (Far vs. Close) interaction effect was found, *F* (2, 80) = 0.096, *p* = 0.909, $\eta {}_{p}^{2}$ 0.002.

**FIGURE 5:**
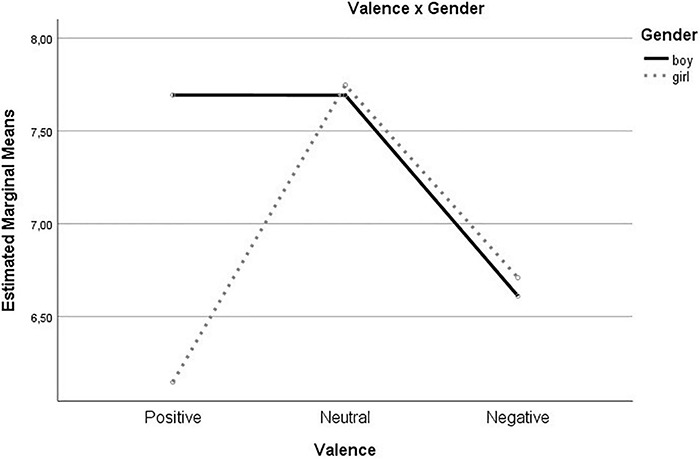
Valence × Gender interaction. Note: estimated marginal means for the valence (Positive vs Neutral vs Negative) × Gender interaction on the number of correctly localized target stimuli are displayed on the *y*-axis.

Next, we added age and gender to the model in order to check whether these variables significantly affected the overall task performance. First, a significant Valence (Positive vs. Neutral vs. Negative) × Gender interaction was found, *F* (2, 76) = 4.86, *p* = 0.010, $\eta_{p}^{2}$ 0.113, indicating that accuracy rates were higher for boys (*M* = 7.69, *SE* = 1.09) compared to girls (*M* = 6.147, *SE* = 1.12) when the target stimulus was positive (see Figure 5). Second, a significant Distance (Far vs. Close) × Age interaction occurred, *F* (1, 38) = 6.45, *p* = 0.015, $\eta_{p}^{2}$ 0.145, revealing that age moderated the effect of distance on accuracy. Gender or age were implied in the 3 Valence (Positive vs. Neutral vs. Negative) × 2 Distance (Far vs. Close), all *p*s > 0.188.

### Main Analyses

#### Visual Attentional Breadth and Emotion Regulation Strategies

The effects pertaining to the self-reported ER strategy variables (FEEL-KJ-DIS, FEEL-KJ-CR, FEEL-KJ-RUM) in the 3 Valence (Positive vs. Neutral vs. Negative) × 2 Distance (Far vs. Close) revealed a significant Distance (Far vs. Close) × FEEL-KJ-DIS, *F* (1, 37) = 5.09, *p* = 0.030, $\eta_{p}^{2}$ = 0.121 interaction. Distraction was associated with visual attentional narrowing when the target stimuli were presented far. No further effects were found.

#### *Post-hoc* Analyses

A paired-samples *t*-test was conducted to examine fluctuations in affect before and after completing the experimental task. There was a significant difference in frustration before (*M* = 21.88, *SD* = 32.32) versus after (*M* = 38.38, *SD* = 42.80) the task; *t*(39) = –3,894, *p* < 0.05, in anger before (*M* = 12, *SD* = 24.75) versus after (*M* = 25.50, *SD* = 37.59) the task; *t*(39) = –2,996, *p* < 0.05 and in boredom before (*M* = 50.50, *SD* = 40.59) versus after (*M* = 63.00, *SD* = 39.04) the task; *t*(39) = –2,492, *p* < 0.05. Suggesting that youth were left significantly more frustrated, angry and bored after completing the ABT.

## Discussion

The current study examined the relationship between the general use of specific ER strategies (i.e., cognitive reappraisal, rumination, and distraction) that rely on attentional deployment and visual attentional breadth for negative, positive, and neutral emotional information in youth at risk for developing psychopathology. To measure ones’ specific ER strategies on a trait level, the FEEL-KJ was used (Braet et al., 2013). To measure visual attentional breadth for emotional information a previously validated Attentional Breadth Task was included [ABT; (Bosmans et al., 2009; Grol and Raedt, 2014; Grol et al., 2015; Van Beveren et al., 2020)]. Three main findings regarding our proposed aims can be reported. First, no evidence was found for the association between trait “cognitive reappraisal” and attentional *broadening* for emotional information. Second, no evidence was found for the relationship between trait “rumination” and attentional *narrowing* for emotional information. Third, trait “distraction” was associated with overall visual attentional narrowing for emotional information. These rather unexpected findings indicate a multifaceted relationship between ER and visual attentional breadth for emotional information. Future research, especially in younger age groups, is needed to further elaborate on these findings.

In the current study, the role of attention was approached by the broaden-and-build theory in which it is stated that negative and positive emotions have different effects on the attentional scope (Fredrickson, 1998; Fredrickson and Joiner, 2002; Fredrickson and Branigan, 2005). Positive emotions are theorized to be related to a broadened attentional scope and greater emotional wellbeing, whereas negative emotions are assumed to be associated with a narrowed attentional scope and risk for psychopathology (Fredrickson and Joiner, 2002). Given that attention is implied in the ER process and ER strategies have the possibility to influence both positive and negative affect, an association between ER and one’s visual attentional scope for emotional information was expected (Aspinwall, 1998; Fredrickson and Joiner, 2002).

The first aim of the current study was to examine the relationship between the adaptive ER strategy “cognitive reappraisal” and visual attentional breadth for emotional information. In general, adaptive ER strategies are associated with the upregulation of positive and downregulation of negative affect. Based on the broaden-and-build theory of positive emotions, it was therefore expected that adaptive ER strategies such as cognitive reappraisal are associated with the broadening of the visual attention (Fredrickson, 2001; Fredrickson and Branigan, 2005). Again, no evidence was found for our hypothesis. In contrast to our study, recent studies clearly distinguish between two separate goals of cognitive reappraisal when researching this strategy (i.e., increasing positive versus decreasing negative affect). Although both goals lead to a reduction in negative affect, a significant difference in the increase of *positive* affect is reported by previous studies that examined this ER strategy form a *state* perspective (McRae et al., 2008, 2012). Unfortunately, the distinct goals of cognitive reappraisal were not assessed by the FEEL-KJ questionnaire, which measures “cognitive reappraisal” as a trait. Therefore, it is difficult to understand *if* and *how* youth used this specific strategy during the completion of the ABT lab task.

The second aim of the current study was to investigate the relationship between the maladaptive ER strategy “rumination” and visual attentional breadth for emotional information. Rumination is more likely to occur in a negative mood and can even reinforce the intensity of the emotions experienced (Nolen-Hoeksema et al., 2008). We hypothesized [based on (Fang et al., 2017)] that the use of “rumination” would be associated with a more narrowed attentional scope. In contrast to existing evidence in adults in which this association was confirmed (e.g., Grol et al., 2015), the current study found no significant relationship between rumination and attentional narrowing in youth for either positive, negative of neutral stimuli on the ABT. A potential explanation for our lack in findings could be that narrowed attention is associated with the use of trait rumination for self-related information (i.e., one’s own feelings and problems) rather than other-related information (Whitmer and Gotlib, 2013; Grol et al., 2015). Although the current study uses trait rumination as an independent variable, no self-related stimuli were included during the experimental task. This could be an interesting avenue for future research.

Finally, we explored the relationship between distraction and visual attentional breadth for emotional information. Traditionally, distraction was categorized as an adaptive ER strategy in previous research (Sheppes et al., 2011) and also in the FEEL-KJ questionnaire this strategy was allocated to the adaptive ER subscale (Cracco et al., 2015). However, more recently researchers revealed a rather ambiguous relation between distraction and emotional well-being (Gratz and Tull, 2011; Craske and Barlow, 2014). Depending on whether it is used in a flexible way to redirect attention or exclusively to avoid negative affect, this strategy is considered adaptive or maladaptive, respectively (Gross, 1998; Van Dillen and Papies, 2015; Wolgast and Lundh, 2017). Because of this conflicting evidence, we hypothesized the general (trait) use of this strategy could be related to both attentional narrowing and attentional broadening for emotional information. Results revealed that distraction was related to lower accuracy rates for the central Emoji (i.e., positive, negative or neutral emoji) when the target stimuli were presented far versus close. This finding suggests that youth scoring high on trait distraction show a general attentional *narrowing* toward emotional information. Such pattern of overall narrowing can be interpreted as maladaptive in the long term since studies in adults found robust evidence for a relationship between attentional narrowing and negative affect, as well as decreased emotional well-being (Fredrickson and Joiner, 2002; Fredrickson and Branigan, 2005).

In addition to our main findings, preliminary analyses indicated that accuracy rates were higher for boys compared to girls when the central Emoji was positive. A possible explanation for this finding can be found in the fact that the adolescence is a critical developmental period regarding reactivity toward emotional information. Simultaneously, gender differences occur in the way this reactivity is handled (Hyde et al., 2008). More specifically, responses to positive affect decrease in adolescent girls compared to boys within the same age-group (Mezulis et al., 2004). Together with this shift, prevalence rates of adolescent depression become higher in girls compared to boys throughout adolescence.

So far, the only study in youth on the relationship of ER and visual attentional breadth for emotional information with the ABT (Van Beveren et al., 2020) found an association between the general use of adaptive ER and attentional broadening for neutral stimuli *in youth within the general population*, suggesting that this could be an indicator of resilience (Isen, 2000; Wadlinger and Isaacowitz, 2006; Sheppes and Gross, 2011). Although we could not replicate these findings, it extends this work to a sample of children and adolescents at risk for psychopathology that commonly demonstrate emotion dysregulation. The current study therefore provides a first and preliminary step in unraveling the different associations between ER strategy use and attentional breadth in at risk groups, throwing more/better light on underlying processes that contribute toward risk and psychopathology.

## Strengths and Limitations

To our knowledge, the current study was the first to investigate the relationship between ER and visual attentional breadth for emotional information in youth at risk for developing psychopathology. Despite the innovativeness and specificity of the current study, several limitations should be acknowledged.

First, the lack of significant results for both rumination and cognitive reappraisal could be due to the small sample size (Kline, 2017). Power analyses were specifically determined for the large intervention study in which the current study was embedded and did not anticipate on the proportion of invalid data on the ABT. Out of 56 participating children and adolescents, 15 were excluded from the current study because of high error rates (Grol and Raedt, 2014). One possible explanation for the level of drop out could be due to the nature of the sample (i.e., youth with subclinical depressive symptoms), the duration of the task (± 40 min), task difficulty and the uncomfortable posture the children and adolescents were placed in. We therefore evaluated the performances on the ABT on several secondary parameters. A closer inspection of the fluctuations in affect before and after the task reveals an increase in feelings of frustration, anger, and boredom after completing the task, reflecting the amount of effort and perseverance the completion of the task required. Yet, previous studies with this task evaluated the ABT as valid, reliable, and doable and the analyses pertaining to task performance in the current study affirm that children and adolescents were able to conduct the task. Perhaps the stimuli used in the current study could have somewhat blurred our findings as the ABT (Bosmans et al., 2009; Grol et al., 2015) was originally developed and validated using faces as central stimuli.

Second, research shows that attentional processes for emotional information may be particularly disturbed in emotional disorders (Gasper and Clore, 2002; Yoon et al., 2015; Gu et al., 2017). For a better understanding of the role of visual attentional breadth for emotional information in the development of psychopathology in youth, the current study conducted in a sample of children and adolescents at risk for developing psychopathology can be considered a valuable pilot study. Future studies, also in clinical samples (i.e., depressed and anxious youth) are designated. Furthermore, as the present sample includes youth with obesity, the findings on the maladaptive role of distraction may be disorder specific. We cannot rule out that also other mechanisms (e.g., impaired self-regulation) that are typically observed in children and adolescents with obesity may have driven the results (Graziano et al., 2010; Golan and Bachner-Melman, 2011; Mehl et al., 2017).

Third, as the current study is cross-sectional, future longitudinal studies with multiple measuring points are crucial for determining the causality and directionality of the relationship between ER and visual attentional breadth for emotional information.

Importantly, we only examined whether self-reported use of three trait ER strategies could be related to visual attentional breadth for emotional information. We did not assess *whether* and *how* participants used these ER strategies while conducting the ABT. Therefore, no conclusions can be drawn about the role of attentional processes during the actual employment of these ER strategies.

Finally, the general use of specific ER strategies is measured through self-report. Using a multi-informant approach could counteract biases for social desirability as well as a lack of introspection in youth, by avoiding all outcome variables to depend on the same method and information source (Kline, 2017). The parent-report version of the FEEL-KJ questionnaire has recently been validated (Van Beveren et al., 2020) and could be of value to include in future studies.

## Future Research and Clinical Implications

As mentioned above, no statement can be made about which ER strategies children and adolescents used during the ABT. Yet, such information would have been of great value for a more thorough understanding of the ER process. A growing body of research (e.g., Sheppes, 2014) suggests that, besides individual differences in the selection of certain strategies, there could be significant differences in effectiveness when using one of these strategies in a specific context. This line of reasoning could explain why certain ER strategies can be both adaptive and maladaptive depending on the context in which they are used. Whereas we already discussed the possibility that distraction can be both adaptive and maladaptive, as evidenced by various studies [e.g., (Wolgast and Lundh, 2017)], cognitive reappraisal has also been critically approached in recent research (Ford and Troy, 2019). More specifically, it is suggested that cognitive reappraisal can be successfully used and still be maladaptive when it is not in line with an individual’s goals and motivation (e.g., reducing feelings important to your identity neglecting your authentic self) or when it is used in the “wrong” situation (e.g., reducing fear in an actual dangerous situation) (Tamir, 2016). Furthermore, cognitive reappraisal can be used unsuccessfully when emotions are too intense or too unfamiliar to generate an effective reappraisal (e.g., not enough reappraisal sources) (Sheppes and Meiran, 2007). Similar findings are found regarding rumination (Joormann et al., 2006). On the one hand, rumination can be considered maladaptive when it entails “brooding,” which refers to making a passive comparison of the current situation with an unachieved standard (e.g., analyzing your own emotions and behavior focusing on the negative) (Treynor et al., 2003). On the other hand, rumination can be adaptive when it contains reflective pondering and thus an analysis of the situation and its causes in a neutral way (e.g., neutrally analysing why you are feeling a certain way in a certain situation) (Cristea et al., 2013). Although the current ER questionnaire (i.e., FEEL-KJ) is a reliable and valid instrument (see Cracco et al., 2015) to measure trait adaptive and maladaptive ER strategies (i.e., based on their factor structure and association with emotional well-being), future research could add experimental studies that start approaching ER strategies using a broader framework for conceptualizing, categorizing and assessing adaptive and maladaptive ER.

Furthermore, besides difficulties in categorizing ER strategies in to maladaptive and adaptive, more recent research suggests it is also too black-and-white to say that all negative emotions narrow and all positive emotions broaden the attentional scope. On the contrary, the impact of positive and negative affect on attentional scope could be more complex and/or flexible than first thought (Huntsinger, 2013). Studies show that not the valence of the affect but rather the motivational intensity (i.e., how important the affect is for an individual) will narrow/broaden the attentional scope (i.e., the higher the motivational intensity, the narrower the attentional scope) (Gable and Harmon-Jones, 2010; Harmon-Jones et al., 2013). Future research could therefore include how personally relevant emotional information is during an attentional breadth task.

Next, some limitations regarding the experimental task (ABT) have already been mentioned. Future studies could also consider using *a global/local processing task* to measure attentional breadth since this task has some merits and has been evaluated across studies (Navon, 1977; Srinivasan and Hanif, 2010). During this type of task participants identify whether the local (narrow) or global (broad) element of a certain stimulus is more salient to them (Sung and Yih, 2016). Based on the limitations of the current study we suggest integrating the strengths of both tasks as well as to (1) include emotional stimuli, (2) implement a standardized mood induction to evoke the use of state ER, and (3) use a survey that measures both the general (trait) and the actual (state) use of ER strategies (during the task).

From a clinical perspective, upcoming research on training ER provides evidence that learning to use specific adaptive ER strategies improves the ability to influence affect (i.e., increasing positive and decreasing negative emotions) (Southam-Gerow, 2013; Berking and Lukas, 2015; Wante et al., 2018). In the current study we hypothesized that ER and visual attentional breadth for emotional information to be associated (Fredrickson, 2001). If any significant relations would have been found between cognitive reappraisal and attention, this would have indicated that training adaptive ER strategies could also broaden the attentional scope of the individual. As both adaptive ER and attentional broadening are considered protective factors, these two mechanisms could potentially reinforce each other and eventually improve mental health (Fredrickson and Joiner, 2002; Rowe et al., 2007; Aldao and Nolen-Hoeksema, 2010, 2012; Gu et al., 2017). However, based on the results of the current study, no assumptions can be made.

## Final Conclusion

To our knowledge, the current study is the first to investigate the relationship between specific emotion regulation strategies and visual attentional breadth in youth at risk for developing psychopathology. Based on the broaden-and-build theory of positive emotions, it was hypothesized that adaptive ER facilitates positive affect and is therefore related to attentional broadening for emotional information. In contrast, given that maladaptive ER maintains negative affect, it was hypothesized that there would be an association with attentional narrowing (Fredrickson, 1998, 2001; Fredrickson and Branigan, 2005). Three emotion regulation strategies that rely on attentional deployment (i.e., cognitive reappraisal, rumination and distraction) were selected and a validated visual attentional breadth task was used. No evidence was found for the proposed associations between both the adaptive ER strategy cognitive reappraisal and the maladaptive ER strategy rumination and visual attentional breadth for emotional information in at risk children and adolescents. However, a remarkable association between the use of distraction and overall visual attentional narrowing toward negative emotional information was found. These results emphasize the ambiguous character of distraction as an ER strategy (e.g., in some contexts it can be considered adaptive but in others maladaptive) and help to further characterize it by suggesting this strategy is predominantly used maladaptively in children and adolescents at risk for psychopathology. These findings indicate the complex relationship between ER and visual attentional breadth and highlight the need for further research in both clinical and larger (selected) samples of youth.

## Data Availability Statement

The raw data supporting the conclusions of this article will be made available by the authors upon reasonable request.

## Ethics Statement

The studies involving human participants were reviewed and approved by Committee of Medical Ethics UZ Gent. Written informed consent to participate in this study was provided by the participants’ legal guardian/next of kin.

## Author Contributions

EB collected all the data. EB and M-LV analyzed the data. All authors were involved in writing the manuscript and had final approval of the submitted and published versions.

## Conflict of Interest

The authors declare that the research was conducted in the absence of any commercial or financial relationships that could be construed as a potential conflict of interest.

## Publisher’s Note

All claims expressed in this article are solely those of the authors and do not necessarily represent those of their affiliated organizations, or those of the publisher, the editors and the reviewers. Any product that may be evaluated in this article, or claim that may be made by its manufacturer, is not guaranteed or endorsed by the publisher.

## Footnotes

1 The General Data Protection Regulation (GDPR) implies the protection of natural persons with regard to the processing of personal data and the free movement of such data.
